# Perceptions of Health Care–Associated Infection Metrics by Infection Control Experts

**DOI:** 10.1001/jamanetworkopen.2023.8952

**Published:** 2023-04-19

**Authors:** Gregory M. Schrank, Westyn Branch-Elliman, Surbhi Leekha, Jonathan Baghdadi, Lisa Pineles, Anthony D. Harris, Daniel J. Morgan

**Affiliations:** 1 Department of Medicine, University of Maryland School of Medicine, Baltimore; 2 Department of Epidemiology and Public Health, University of Maryland School of Medicine, Baltimore,; 3 Section of Infectious Diseases, VA Boston Healthcare System, Boston, Massachusetts; 4 Harvard Medical School, Boston, Massachusetts

## Abstract

This survey study examines the perceptions of US infection control experts on commonly reported measures of health care–associated infections.

## Introduction

Clinicians and infection control (IC) experts have raised concerns about the validity and impact of public reporting of health care–associated infection (HAI) on clinical care.^1,2^ Time spent collecting, processing, and responding to publicly reported HAIs may limit other IC activities and patient safety.^1^ We conducted a survey of US IC experts to evaluate perceptions of commonly reported HAI measures.

## Methods

This survey study was determined to be non–human participants research by the University of Maryland, Baltimore institutional review board and did not require informed consent because it used nonidentifiable data, in accordance with 45 CFR §46. This study followed the American Association for Public Opinion Research (AAPOR) reporting guideline. From October to December 2019, 71 US institutions in the Society for Healthcare Epidemiology of America Research Network were invited to complete a web-based survey. Survey respondents were provided with a gift card for successful completion of the survey. Data analysis was performed using Excel, version 2012 (Microsoft) in January 2021. Facility demographics and characteristics were collected as part of the survey. Likert scale questions assessed agreement with statements about each HAI metric, and metrics were ranked for benefits and value of public reporting. Additional questions assessed the respondents’ perception of the impact of metric reporting on other IC activities.

HAI metrics included surgical site infection (SSI), infection-related ventilator-associated condition (IVAC), the Sepsis Early Management Bundle (SEP-1), central line−associated bloodstream infection (CLABSI), catheter-associated urinary tract infection (CAUTI), *Clostridioides difficile* infection (CDI), and methicillin-resistant *Staphylococcus aureus* bloodstream infection (MRSA BSI). These metrics are all mandated for reporting except for IVAC, which varies by state or territory. Most metrics are publicly reported and tied to reimbursement.^1,2,3^

## Results

Of the 71 institutions given the survey, IC experts at 43 institutions (60.5%) completed it. Facility and respondent characteristics are described in the Table. Perceptions of HAI metrics are presented in the Figure. Respondents perceived that MRSA BSI (40 respondents [95%]) and SSI (36 respondents [88%]), but not IVAC (6 respondents [15%]) and SEP-1 (5 respondents [12%]) identified true infection. Except for SEP-1 and IVAC, respondents perceived all metrics as being responsive to prevention efforts.

**Table.** Characteristics of Facilities and Respondents to Society for Healthcare Epidemiology of America Research Network Survey

Characteristic	Respondents, No. (%)
Facilities	
Region of the US	
Northeast	11 (26)
Midwest	10 (23)
South	13 (30)
West	9 (21)
Type	
Academic teaching hospital	29 (67)
Community hospital	9 (21)
Veteran’s Affairs hospital	3 (7)
Public health or other	2 (5)
Bed size	
<500	20 (47)
500-750	10 (23)
751-1000	9 (21)
>1000	4 (9)
Hospital epidemiologists, median (IQR), No.	1 (1-2)
Infectious preventionists, median (IQR), No.	5 (3-7)
Respondent role	
Health care epidemiologist	35 (81)
Antimicrobial stewardship physician	3 (7)
Infection preventionist	2 (5)
Infectious disease physician administrator	2 (5)

**Figure.**
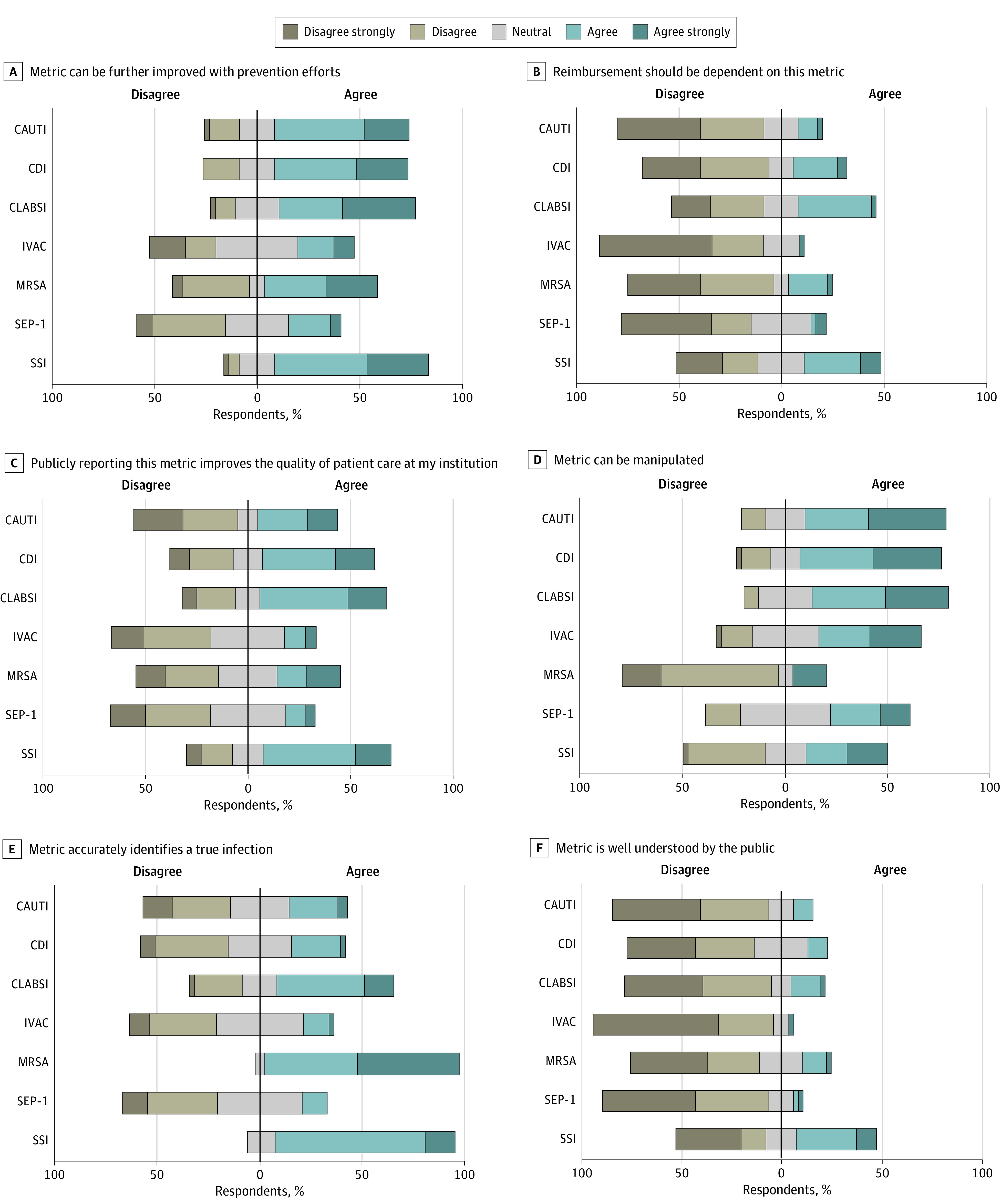
Perceptions of Infection Control Experts on Commonly Measured Health Care–Associated Infections The median neutral response is set at zero (vertical axis) to demonstrate the degree of agreement or disagreement with each statement. CAUTI indicates catheter-associated urinary tract infection; CDI, *Clostridioides difficile* infection; CLABSI, central line-associated bloodstream infection; IVAC, infection-related ventilator-associated condition; MRSA BSI, methicillin-resistant *Staphylococcus aureus* bloodstream infection (MRSA BSI); SEP-1, sepsis Centers for Medicare & Medicaid Services CMS Core Measure; SSI, surgical site infection.

Negative aspects of HAI metrics included that most were perceived as being susceptible to manipulation; CAUTI (29 respondents [69%]), CDI (29 respondents [69%]), and CLABSI (28 respondents [67%]) were considered most susceptible. Similarly, 36 respondents (84%) believed "hospitals and/or staff intentionally manipulate (game) publicly reported HAI rates." No metrics were perceived to be well understood by the public, and respondents did not believe any metrics should be linked to hospital reimbursement. Additionally, 35 respondents (81%) believed compliance with the SEP-1 metric leads to unnecessary antimicrobial use. Respondents identified SSI (30 respondents [75%]), CLABSI (28 respondents [67%]), and CDI (26 respondents [65%]) as most important for improving care and IVAC (6 respondents [15%]) and SEP-1 (6 respondents [15%]) as least important for quality of care. A majority of respondents (41 respondents [95%]) believed that efforts focused on HAI metrics limited other infection prevention activities.

## Discussion

In this survey study of US IC experts, many respondents perceived that HAI quality metrics do not accurately reflect underlying clinical infections and are not well understood by the public. We also identified a strong perception that HAI metrics can be manipulated and should not be tied to financial reimbursement. Most respondents thought efforts directed toward collecting and publicly reporting metrics distracted from infection prevention activities. Distraction from reporting metrics may be improved by current efforts by federal officials to automate and move to laboratory-based HAI metrics.

The IC experts in our survey perceived positives to public reporting, including a broad sense that this requirement brought additional resources and support to IC at their institutions. Metrics for CLABSI and SSI were seen as beneficial, whereas IVAC and SEP-1 metrics were perceived as not beneficial, in particular for public reporting, consistent with suggestions by others.^1,4,5^ CAUTI, MRSA BSI, and CDI metrics were considered of uncertain value. Federal officials should consider which HAI metrics are highest value when deciding to tie metrics to public reporting and reimbursement.^6^ Limitations of this study include that the majority of respondents were infection control experts at academic hospitals, and survey results may not reflect the opinions of all HAI public reporting stakeholders.

## Notes

Supplement.
Data Sharing Statement
