# Supplementary material for: Perceptions of Health Care–Associated Infection Metrics by Infection Control Experts
Source: JAMA Netw Open. 2023 Apr 19;6(4):e238952. doi: 10.1001/jamanetworkopen.2023.8952 (PMC10116362; doi:10.1001/jamanetworkopen.2023.8952)
Supplement: Supplement. — Data Sharing Statement [file jamanetwopen-e238952-s001.pdf]

Data Sharing Statement

Schrank. Perceptions of Health care-Associated Infection Metrics by Infection Control Experts. *JAMA Netw Open*. Published April 19, 2023. doi:10.1001/jamanetworkopen.2023.8952

Data
Data available: No
